# Epiregulin, epigen and betacellulin antibodies and axial elongation in young guinea pigs with lens-induced myopization

**DOI:** 10.1186/s12886-022-02417-8

**Published:** 2022-04-27

**Authors:** Li Dong, Rui-Heng Zhang, Wen-Da Zhou, Yi-Fan Li, He-Yan Li, Hao-Tian Wu, Xu-Han Shi, Jost. B. Jonas, Wen-Bin Wei

**Affiliations:** 1 Beijing Tongren Eye Center, Beijing Key Laboratory of Intraocular Tumor Diagnosis and Treatment, Beijing Ophthalmology & Visual Sciences Key Lab, Medical Artificial Intelligence Research and Verification Key Laboratory of the Ministry of Industry and Information Technology, Beijing Tongren Hospital, Capital Medical University, Beijing, China; 2 Beijing Institute of Ophthalmology and Beijing Ophthalmology and Visual Science Key Lab, Beijing Tongren Eye Center, Beijing Tongren Hospital, Capital Medical University, Beijing, China; 3 Department of Ophthalmology, Medical Faculty Mannheim, Heidelberg University, Mannheim, Germany; 4 Institute of Molecular and Clinical Ophthalmology Basel, Basel, Switzerland; 5 Privatpraxis Prof Jonas Und Dr Panda-Jonas, Heidelberg, Germany

**Keywords:** Myopia, Epidermal Growth Factor, Axial Length, Epiregulin, Epigen, Betacellulin, Experimental Myopia, Ocular Elongation

## Abstract

**Background:**

To examine an effect of intravitreally applied antibodies against epidermal growth factor family members, namely epiregulin, epigen and betacellulin, on ocular axial elongation.

**Methods:**

The experimental study included 30 guinea pigs (age:3–4 weeks) which underwent bilateral lens-induced myopization and received three intraocular injections of 20 µg of epiregulin antibody, epigen antibody and betacellulin antibody in weekly intervals into their right eyes, and of phosphate-buffered saline into their left eyes. Seven days after the last injection, the animals were sacrificed. Axial length was measured by sonographic biometry.

**Results:**

At baseline, right eyes and left eyes did not differ (all *P* > 0.10) in axial length in neither group, nor did the interocular difference in axial length vary between the groups (*P* = 0.19). During the study period, right and left eyes elongated (*P* < 0.001) from 8.08 ± 0.07 mm to 8.59 ± 0.06 mm and from 8.08 ± 0.07 mm to 8.66 ± 0.07 mm, respectively. The interocular difference (left eye minus right eye) in axial elongation increased significantly in all three groups (epiregulin-antibody:from 0.03 ± 0.06 mm at one week after baseline to 0.16 ± 0.08 mm at three weeks after baseline;*P* = 0.001); epigen-antibody group:from -0.01 ± 0.06 mm to 0.06 ± 0.08 mm;*P* = 0.02; betacellulin antibody group:from -0.05 ± 0.05 mm to 0.02 ± 0.04 mm;*P* = 0.004). Correspondingly, interocular difference in axial length increased from -0.02 ± 0.04 mm to 0.13 ± 0.06 mm in the epiregulin-antibody group (*P* < 0.001), and from 0.01 ± 0.05 mm to 0.07 ± 0.05 mm in the epigen-antibody group (*P* = 0.045). In the betacellulin-antibody group the increase (0.01 ± 0.04 mm to 0.03 ± 0.03 mm) was not significant (*P* = 0.24).

**Conclusions:**

The EGF family members epiregulin, epigen and betacellulin may be associated with axial elongation in young guinea pigs, with the effect decreasing from epiregulin to epigen and to betacellulin.

## Introduction

Axial elongation of the ocular globe is a hallmark of axial myopization. The mechanisms leading to an increased sagittal diameter of the eye have remained elusive so far. Anatomical studies have revealed that during the process of axial elongation the eye changes its shape from a sphere in the state of emmetropia to a prolate configuration in the state of axial myopia [1, 2]. Correspondingly, the coronal eye diameters in the horizontal and vertical direction increase by only 0.10 to 0.20 mm for each mm increase in axial length [2]. Studies also showed, that the choroid and sclera decrease in thickness, most pronounced at the posterior pole and least marked in the ora serrata region or anterior to it. The thickness of Bruch´s membrane was not associated with axial elongation [1–6]. The thickness of the retina and of the choriocapillaris and thickness and density of the retinal pigment epithelium (RPE) cells at the posterior pole were not correlated with axial length, while the retinal thickness and RPE cell density in the midperiphery of the eye density decreased with longer axial length [6–8]. The axial elongation-associated increase in the disc-fovea distance was due to the development and enlargement of Bruch´s membrane free parapapillary gamma zone in the temporal parapapillary region [9]. These observations on the anatomical changes in axial myopia, in particular the thinning of the choroid in the macular region, led to the notion, that an enlargement of Bruch´s membrane in the equatorial region leads to a sagittal elongation of the eye, with a secondary compression and thinning of the posterior choroid and secondary thinning of the sclera, most marked at the posterior pole [6]. With Bruch´s membrane M being produced by the RPE, a hypothesis was formed that a messenger molecule makes the midperipheral RPE produce and enlarge Bruch´s membrane. In search of the such a messenger molecule, previous investigations suggested that the epidermal growth factor (EGF) and its family members may be potential candidates [10–12]. The studies revealed that guinea pigs with or without lens-induced axial elongation showed a reduction in axial elongation if antibodies against EGF, the EGF receptor, and against the EGF family members amphiregulin and neuregulin-1 were intravitreally injected. As a corollary, intravitreally injected EGF and EGF family members were associated with an increase in axial elongation [10–12]. Since the EGF family is heterogenous, we examined here whether other EGF family members, notably epiregulin, epigen, and betacellulin may be involved in the process of axial elongation including the vitreous cavity elongation. With EGF, amphiregulin and neuregulin-1 already included in previous studies, we chose the EGF family members epiregulin, epigen, and betacellulin as test molecules in the present study to get an overview over the whole EGF family in its potential relationship with ocular axial elongation. Epigen and epiregulin, besides amphiregulin, are low-affinity ligands, while betacellulin, besides EGF, transforming growth factor-a, and heparin binding EGF-like growth factor, is a high-affinity ligand.

## Methods

The experimental investigation consisted of 30 guinea pigs with an age of three to four weeks and with a mean body weight of 100–150 g at baseline. The Ethics Committee of the Beijing Tongren Hospital study approved the study, which followed the ARVO Statement for the use of animals in ophthalmic and vision research. All animals were kept at a constant temperature of 26 °C, and food and water were supplied regularly. The animals were housed under a 12-h light/dark cycle (automatically on/off at 8 am and 8 pm) with a light intensity of 500 Lux. The animals were randomly assigned to three study groups, each including 10 animals. In these three study groups, the animals received three intravitreal injections of antibodies against epiregulin (MAB1068, R&D Systems, Bio-Techne Co., Minnesota, USA), epigen (MAB11271, R&D Systems, Bio-Techne Co., Minnesota, USA), and betacellulin (AF1025, R&D Systems, Bio-Techne Co., Minnesota, USA), respectively, in a dose of 20 µg into their right eyes in weekly intervals. The dose of 20 µg was chosen since in previous investigations with similar study designs, doses of 20 µg of antibodies against amphiregulin and neuregulin1 as other EGF family members had been used [10–12]. The injections were performed at baseline, and at one and two weeks after baseline. As described in detail recently, we used a Hamilton microsyringe (HamiltonI® Microliter™ syringe, Sigma-Aldrich, St. Louis, MO, USA) to carry out the injections with an injection volume of 5 μL [10–12]. The left eyes received intravitreal injections of phosphate-buffered saline with the same injection volume of 5 µL (#75,889, USB Co., Cleveland, OH, USA). All injections were carried out 2 mm posterior to the corneal limbus, and the injection needles were aimed towards the posterior pole.

As also described in detail previously, all animals underwent bilateral negative lens-induced myopization [10–12]. Negative lens-induced myopization is besides form-deprivation induced myopia one of the possibilities to externally induce a myopic axial elongation in animals such as chicks, guinea pigs, tree shrews, primates and mice [13, 14]. Negative lens-induced myopization is a closed loop condition, in which axial elongation decreases the hyperopic defocus caused by the lens and in which the axial elongation stops when the growth stimulus has been neutralized. When the lens is removed, the condition can be (partially) reversible depending on the age of the animal and other conditions. At baseline, goggles with a refractive power of -10.0 diopters were glued onto the orbital rims of both eyes, so that the animals could freely open their eyes and blink. The animals were daily examined to check that the googles were clean and in place. For the re-injections, the goggles were removed, biometric and ophthalmoscopical examinations were repeated, ofloxacin eye drops (Santen Co., Osaka, Japan) were applied, and the injection was carried out. After re-application of ofloxacin eye drops, the goggles were re-applied. The whole process including removal of the goggles, performing the measurements and re-adaptation of the goggles did not take more than 10 to 15 min.

All animals underwent a series of examinations at each of the injection time points including the baseline time point, and at one week after the last injection. i.e., at day 1, 8, 15 and 22. The total study duration was three weeks. The examinations included ocular sonographic biometry (A/B-mode scan; oscillator frequency: 11 MHz; Quantel Co., Les Ulis, France) performed under topical anesthesia (oxybuprocaine hydrochloride eye drops; Santen Co., Osaka, Japan), to measure the axial length, anterior chamber depth, lens thickness and vitreous cavity length. The sound conducting velocity was assumed to be 1557 m/s for the measurement of the anterior chamber, 1723 m/s for the measurement of the lens and 1540 m/s for the measurement of vitreous chamber. Axial length was defined as the distance between the anterior corneal surface and the surface of the central retina. Care was taken that the cornea was not indented during the sonographic measurements. The sonographic measurements were repeated until a series of 10 consistent ultrasound measurements had been obtained, the standard deviation of which had to be lower than 0.1 mm. We averaged the 10 measurements and took the mean for further statistical analysis.

We additionally measured the intraocular pressure by hand-held tonometry (Tonopen, Reichert Inc, Depew,NY, USA) at 4 o´clock, and the mean values were calculated from three measurements.

Using a commercially available software program (SPSS 27.0; IBM Corp., Armonk, NY, USA), we calculated the mean ± standard deviation of the main outcome parameter, i.e., axial length. We compared the axial length and the amount of axial elongation during the study period between the eyes of the same animals using Student’s t-test for paired samples, while we applied the Student´s t-test for un-paired samples for the comparison of the three antibody groups of epiregulin-antibody, epigen-antibody and betacellulin-antibody. A two-sided *P* < 0.05 was considered statistically significant.

## Results

In the left eyes (control eyes) of all three antibody groups, axial length increased from 8.08 ± 0.07 mm at baseline to 8.66 ± 0.07 mm with an axial elongation of 0.58 ± 0.06 mm. The epiregulin-antibody group (0.61 ± 0.06 mm) and the epigen-antibody group (0.60 ± 0.04 mm) did not differ significantly (*P* = 0.60) in axial elongation, while axial elongation was significantly (*P* = 0.002; ANOVA) lower in the betacellulin-antibody group (0.54 ± 0.03 mm) (Table 1).

**Table 1:** Axial length and axial elongation and vitreous cavity length and its elongation during the study period in young guinea pigs with bilateral lens-induced myopization and with unilateral intravitreal injections (right eyes) of epidermal growth factor family antibodies and contralateral intravitreal injections (left eyes) of phosphate buffered solution (mean ± standard deviations)

Group	n	Eye	Baseline	One week follow-up	Two weeks follow-up	Three weeks follow-up	One week follow-up	Two weeks follow-up	Three weeks follow-up
		Axial length (mm)					Axial length difference to baseline (axial elongation)		
Epiregulin- Antibody	10	Right	8.10 ± 0.09	8.28 ± 0.04	8.40 ± 0.04	8.55 ± 0.05	0.19 ± 0.08	0.30 ± 0.11	0.46 ± 0.06
		Left	8.07 ± 0.08	8.29 ± 0.03	8.46 ± 0.06	8.69 ± 0.06	0.22 ± 0.08	0.39 ± 0.13	0.61 ± 0.06
		*P*-value	0.13	0.60	0.004	< 0.001	0.15	0.004	< 0.001
Epigen- Antibody	10	Right	8.08 ± 0.07	8.31 ± 0.05	8.44 ± 0.04	8.62 ± 0.06	0.24 ± 0.08	0.37 ± 0.09	0.54 ± 0.07
		Left	8.08 ± 0.07	8.31 ± 0.03	8.45 ± 0.07	8.68 ± 0.07	0.23 ± 0.08	0.54 ± 0.07	0.60 ± 0.04
		*P*-value	0.68	0.86	0.86	0.002	0.64	0.92	0.045
Betacellulin- Antibody	10	Right	8.07 ± 0.05	8.31 ± 0.03	8.47 ± 0.04	8.60 ± 0.05	0.24 ± 0.03	0.40 ± 0.05	0.52 ± 0.04
		Left	8.08 ± 0.05	8.28 ± 0.05	8.46 ± 0.06	8.62 ± 0.05	0.19 ± 0.04	0.37 ± 0.09	0.54 ± 0.03
		*P*-value	0.35	0.04	0.41	0.04	0.01	0.22	0.24
		Vitreous cavity length (mm)					Vitreous cavity length difference to baseline (vitreous cavity elongation)		
Epiregulin- Antibody	10	Right	3.40 ± 0.24	3.48 ± 0.11	3.51 ± 0.09	3.61 ± 0.10	0.08 ± 0.23	0.11 ± 0.26	0.20 ± 0.26
		Left	3.45 ± 016	3.49 ± 0.16	3.54 ± 0.05	3.68 ± 0.10	0.05 ± 0.19	0.10 ± 0.15	0.23 ± 0.18
		*P*-value	0.54	0.84	0.26	0.05	0.58	0.80	0.88
Epigen- Antibody	10	Right	3.51 ± 0.17	3.55 ± 0.11	3.47 ± 0.15	3.64 ± 0.07	0.03 ± 0.18	-0.04 ± 0.29	0.13 ± 0.18
		Left	3.49 ± 0.15	3.50 ± 0.18	3.54 ± 0.05	3.71 ± 0.09	0.01 ± 0.18	0.05 ± 0.16	0.23 ± 0.16
		*P*-value	0.48	0.59	0.20	0.11	0.51	0.15	0.09
Betacellulin- Antibody	10	Right	3.49 ± 0.16	3.42 ± 0.11	3.53 ± 0.14	3.68 ± 0.14	-0.07 ± 0.22	0.04 ± 0.27	0.18 ± 0.18
		Left	3.47 ± 0.13	3.47 ± 0.14	3.63 ± 0.18	3.58 ± 0.08	0.00 ± 0.10	0.16 ± 0.14	0.11 ± 0.17
		*P*-value	0.64	0.54	0.31	0.047	0.47	0.14	0.54

At baseline, the right eyes (study eyes) and the left eyes (control eyes) did not differ significantly in axial length in neither group (all *P* > 0.10), nor did the interocular difference in axial length at baseline differ between the groups (*P* = 0.19) (Table 1, 2). During the study period, the right eyes in all groups showed an enlargement (*P* < 0.001). Axial length increased from 8.10 ± 0.09 mm at baseline to 8.55 ± 0.05 mm at study end in the epiregulin-antibody group, from 8.08 ± 0.07 mm to 8.62 ± 0.06 mm in the epigen-antibody group, and from 8.07 ± 0.05 mm to 8.60 ± 0.05 mm in the betacellulin-antibody group (Table 1). The axial elongation in the right eyes during the study period was smaller (*P* = 0.008) in the epiregulin-antibody group (0.46 ± 0.06 mm) than in the epigen-antibody group (0.54 ± 0.07 mm) and the betacellulin-antibody group (0.52 ± 0.04 mm) (Table 1).

**Table 2:** Interocular difference in axial length and in axial elongation in young guinea pigs with bilateral lens-induced myopization and with unilateral intravitreal injections (right eyes) of epidermal growth factor family antibodies and contralateral intravitreal injections (left eyes) of phosphate buffered solution (mean ± standard deviations)

Group	n	Baseline	One week follow-up	Two weeks follow-up	Three weeks follow-up	One week follow-up	Two weeks follow-up	Three weeks follow-up	Difference between one-week follow-up and three-week follow-up *(P*-value)
		Interocular difference in axial length (left eye minus right eye)				Interocular difference in axial elongation (left eye minus right eye)			
Epiregulin- Antibody	10	-0.02 ± 0.04	0.01 ± 0.05	0.07 ± 0.05	0.13 ± 0.06	0.03 ± 0.06	0.09 ± 0.07	0.16 ± 0.08	0.001
Epigen- Antibody	10	0.01 ± 0.05	0.00 ± 0.05	0.00 ± 0.07	0.07 ± 0.05	-0.01 ± 0.06	0.00 ± 0.09	0.06 ± 0.08	0.02
Betacellulin- Antibody	10	0.01 ± 0.04	-0.04 ± 0.05	-0.01 ± 0.04	0.03 ± 0.03	-0.05 ± 0.05	-0.02 ± 0.06	0.02 ± 0.04	0.004
Difference between Antibody Groups (*P*-value (ANOVA))		0.19	0.12	0.01	< 0.001	0.02	0.005	< 0.001	
		Interocular difference in vitreous cavity length (left eye minus right eye)				Interocular difference in vitreous cavity elongation (left eye minus right eye)			
Epiregulin- Antibody	10	0.05 ± 0.15	0.02 ± 0.18	0.03 ± 0.12	0.08 ± 0.12	-0.03 ± 0.23	-0.01 ± 0.22	0.03 ± 0.21	0.17
Epigen- Antibody	10	-0.03 ± 0.12	-0.01 ± 0.20	0.07 ± 0.15	0.07 ± 0.11	-0.02 ± 0.23	0.09 ± 0.21	0.10 ± 0.17	0.07
Betacellulin- Antibody	10	-0.02 ± 0.11	0.05 ± 0.23	0.10 ± 0.24	-0.10 ± 0.15	0.07 ± 0.26	0.12 ± 0.23	-0.07 ± 0.22	0.17
Difference between Antibody Groups (*P*-value (ANOVA))		0.38	0.52	0.72	0.008				

**Fig. 1:**
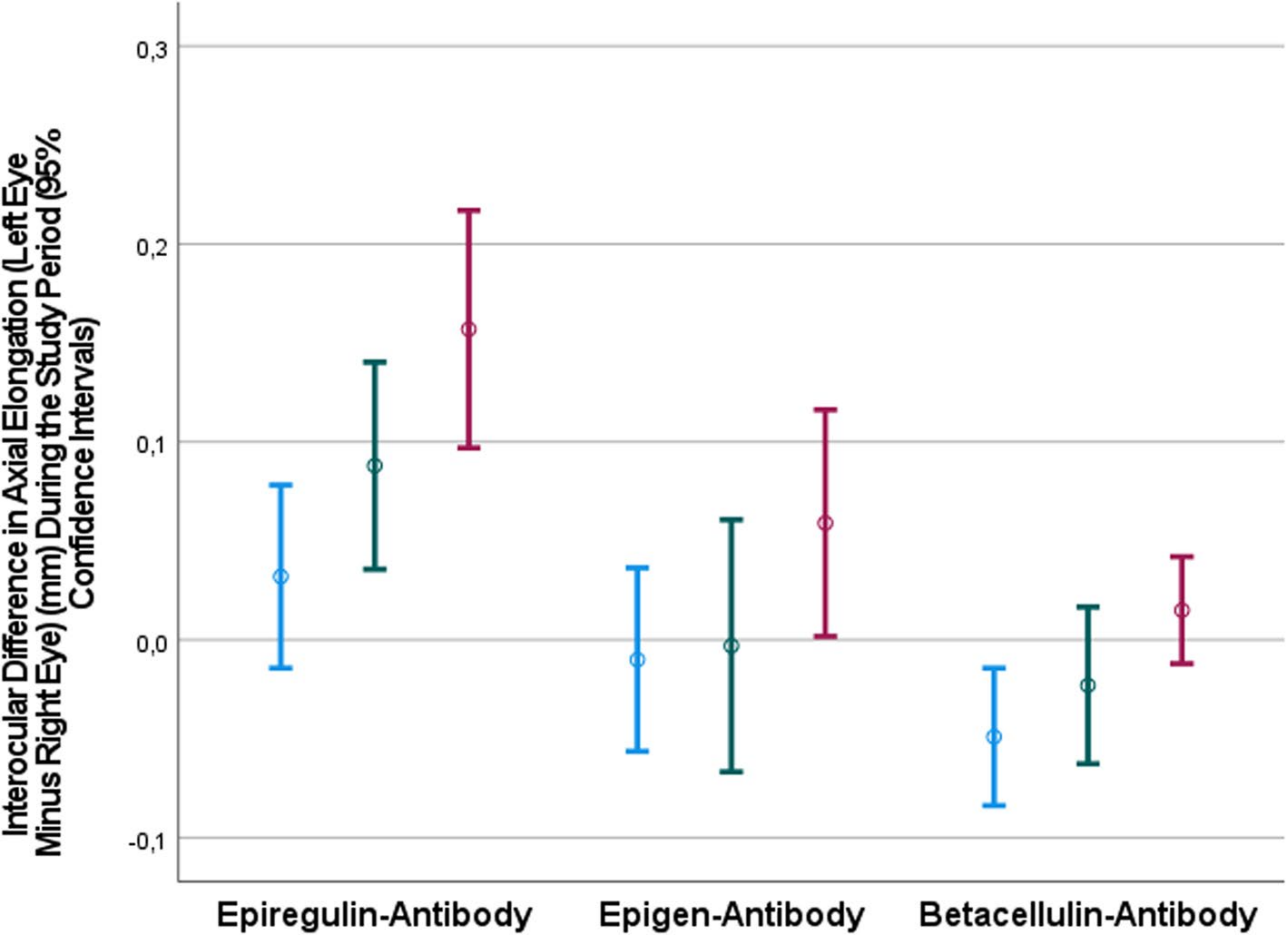
Graph showing the distribution of the interocular difference (left eye minus right eye) in axial elongation during the study period at one week (blue bars), two weeks (green bars) and three weeks (red bars) after baseline in guinea pigs with bilateral lens-induced axial elongation and receiving intravitreal injections of antibodies to epiregulin, epigen and betacellulin

The interocular difference in axial elongation during the study period increased significantly in all three antibody groups, from 0.03 ± 0.06 mm at the first week after baseline to 0.16 ± 0.08 mm at the third week after baseline in the epiregulin-antibody (*P* = 0.001), from -0.01 ± 0.06 mm to 0.06 ± 0.08 mm in the epigen-antibody group (*P* = 0.02), and from -0.05 ± 0.05 mm to 0.02 ± 0.04 mm in the betacellulin antibody group (*P* = 0.004) (Table 2) (Fig. 1).

**Fig. 2:**
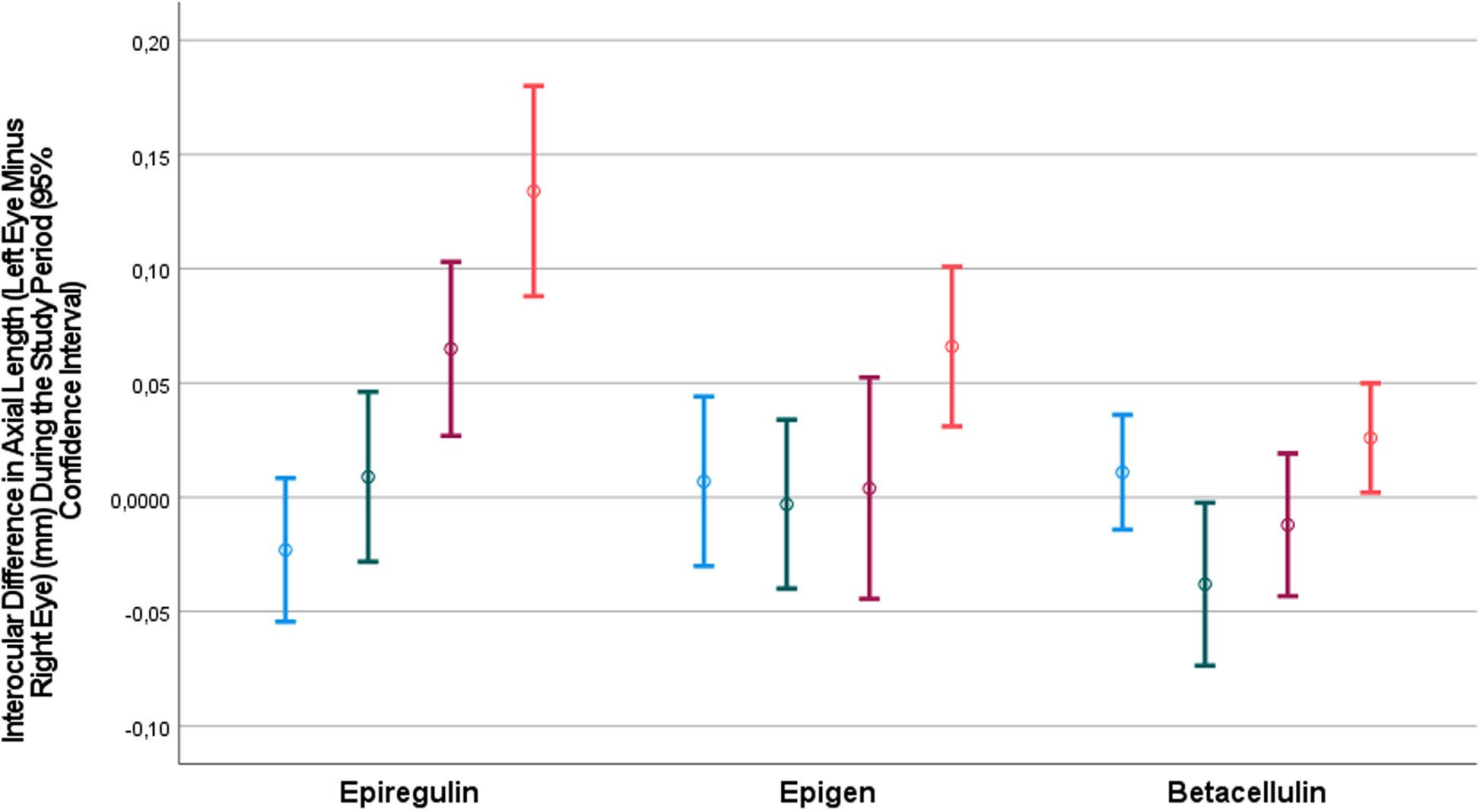
Graph showing the distribution of the interocular difference (left eye minus right eye) in axial length at baseline (blue bars), and at one week (green bars), two weeks (purple bars) and three weeks (orange bars) after baseline, in young guinea pigs with bilateral lens-induced myopization and with unilateral intravitreal injections (right eyes) of epidermal growth factor family antibodies and contralateral intravitreal injections (left eyes) of phosphate buffered solution

In a similar manner, the interocular difference (left eye minus right eye) in axial length increased significantly during the study period, from -0.02 ± 0.04 mm at baseline to 0.13 ± 0.06 mm at the study end in the epiregulin-antibody group (*P* < 0.001), and from 0.01 ± 0.05 mm to 0.07 ± 0.05 mm in the epigen-antibody group (*P* = 0.045) (Fig. 2). The increase from 0.01 ± 0.04 mm to 0.03 ± 0.03 mm in the betacellulin-antibody group was not statistically significant (*P* = 0.24). In the latter group, the increase in the interocular difference in axial length was significant between the first week after baseline and the third week after baseline (from -0.04 ± 0.05 mm to 0.03 ± 0.03 mm; *P* = 0.004) (Table 2) (Fig. 2).

In an inter-group comparison, the increase in the interocular difference in axial length was largest in the epiregulin-antibody group, followed by the epigen-antibody group, and it was smallest in the betacellulin-antibody group (Table 2). As a corollary, the interocular difference in axial elongation was largest in the epiregulin-antibody group, followed by the epigen-antibody group, and eventually the betacellulin-antibody (Table 2) (Fig. 2).

In a similar manner, the right eyes and the left eyes did not differ significantly in vitreous cavity length in neither group (all *P* > 0.40), nor did the interocular difference in vitreous cavity length at baseline differ between the groups (*P* = 0.38) (Table 1, 2). During the study period, the right eyes in all groups showed an enlargement (*P* < 0.001). Vitreous cavity length increased from 3.40 ± 0.24 mm at baseline to 3.61 ± 0.10 mm at study end in the epiregulin-antibody group, from 3.51 ± 0.17 mm to 3.64 ± 0.07 mm in the epigen-antibody group, and from 3.49 ± 0.16 mm to 3.68 ± 0.14 mm in the betacellulin-antibody group (Table 1). The vitreous cavity elongation in the right eyes during the study period did not differ significantly (*P* = 70) between the epiregulin-antibody group (0.20 ± 0.26 mm), the epigen-antibody group (0.13 ± 0.18 mm) and the betacellulin-antibody group (0.18 ± 0.18 mm) (Table 1).

The interocular difference in vitreous cavity elongation during the study period increased, but not significantly in the epigen-antibody group (*P* = 0.17) from -0.03 ± 0.23 mm at the first week after baseline to 0.03 ± 0.21 mm at the third week after baseline, and from -0.02 ± 0.23 mm to 0.10 ± 0.17 mm in the epigen-antibody group (*P* = 0.07), while in the betacellulin antibody group, the value changed from 0.07 ± 0.26 mm at the first week after baseline to 0.12 ± 0.23 mm at the second week after baseline and to -0.07 ± 0.22 mm at study end (*P* = 0.17) (Table 2).

In a similar manner, the interocular difference (left eye minus right eye) in vitreous cavity length increased, but not significantly during the study period, from 0.05 ± 0.18 mm at baseline to 0.08 ± 0.12 mm at the study end in the epiregulin-antibody group (*P* = 0.17), and from -0.03 ± 0.12 mm to 0.07 ± 0.11 mm in the epigen-antibody group (*P* = 0.07) (Table 2).

In the epiregulin-antibody group, the epigen-antibody group and the betacellulin-antibody group, the intraocular pressure (IOP) did not differ significantly between the measurements obtained at baseline and the readings performed at study end (15.9 ± 3.4 mmHg versus 15.5 ± 2.8 mmHg (*P* = 0.50), 14.8 ± 2.9 mmHg versus 14.6 ± 2.9 mmHg (*P* = 0.08) and 15.5 ± 3.0 mmHg versus 15.1 ± 2.2 mmHg (*P* = 0.89), respectively.

None of the eye showed intraocular inflammatory changes or a toxic effect upon intravital ophthalmoscopical examinations.

## Discussion

In this experimental study, blocking the EGF family members epiregulin, epigen and betacellulin resulted in a reduction of axial elongation in young guinea pigs with bilateral lens-induced myopization. The effect was strongest for epiregulin and weakest for betacellulin, the intravitreal application of which was associated with a significant increase in the bilateral difference of axial elongation, while the bilateral difference in axial length was significant only from the first week after baseline onwards (Figs. 1, 2).

These findings concur with results of previous studies showing that intravitreally applied antibodies against other EGF family members, notably antibodies against amphiregulin, EGF and neuregulin-1 as well as antibodies against the EGF receptor, resulted in a dose-dependent decrease of axial elongation in young guinea pigs with, or without, lens-induced axial elongation [10–12]. As a corollary, studies revealed that the intraocularly injected EGF family members, i.e., amphiregulin, EGF and neuregulin-1, resulted in a dose-dependent increase in axial elongation [10–12]. The observations made in our study extend the findings made in the previous studies to additional members of the whole EGF family and further strengthen the notion, that EGF and its family members are associated with the process of axial elongation in guinea pigs.

EGF and its family members may thus belong to the list of molecules which were associated with the development of experimental myopia in previous investigations. The list consists of substances from the cholinergic system like pirenzepine and atropine, dopamine, apomorphine, molecules such as glucagon-like peptide-1, glucagon and insulin, and transforming growth factor beta, basic fibroblast growth factor, sonic hedgehog, nitric oxide, retinoic acid, vasoactive intestinal polypeptide, and molecules in association with the immediate early genes and the FBJ osteosarcoma oncogene [15–24]. It may be of interest, that the EGF receptor can be activated or influenced by the m1-muscarinic acetylcholine receptor, dopamine, vasoactive intestinal peptide and glucagon-like peptide [25–28].

None of the eyes included into this study developed intraocular inflammatory changes. It agrees with observations made in previous investigations in which the intraocular application of antibodies against other EGF family members neither showed an intraocular effect [10–12].

If the EGF family members are associated with the process of axial elongation, the question arises on which mechanisms such an effect may be based upon. Previous histomorphometric and clinical studies showed, that longer axial length was associated with a thinning of the sclera and choroid mostly at the posterior pole, while the thickness of the retina, Bruch´s membrane and choriocapillaris and the density of the retinal pigment epithelium (RPE) cells at the posterior pole was not related with axial length [29]. In contrast, retinal thickness and RPE cell density in the midperiphery of the fundus decreased with longer axial length. The axial elongation-associated increase in the optic disc-fovea distance was found to be due to the development and enlargement of parapapillary gamma zone in the temporal parapapillary region, while the length of Bruch´s membrane in the macular region was not related with axial length [29]. The panoply of these findings led to the hypothesis, that myopic axial elongation is caused mainly by an enlargement of Bruch´s membrane in the midperipheral region of the eye, changing the eye shape from a sphere to a prolate configuration, pushing the posterior Bruch´s membrane backward with a secondary compression and thinning of the choroid and sclera, leading to a decrease in the RPE cell density and retinal thinning in the midperiphery, and a constancy of the retinal thickness and RPE cell density in the macular region [6]. Since Bruch´s membrane is produced by the RPE, the findings of the present study in combination with the observations made in the previous investigations fit with the notion of Bruch´s membrane as the driving structure for axial elongation, since the EGF family members, including epiregulin and epigen, and to a minor degree betacellulin, may stimulate the RPE in the midperiphery. EGF and its family members have been shown to be a driver of the initiation of RPE cell proliferation [30].

It has remained unclear why EGF family members, such as epigen, epiregulin and betacellulin may differ in their influence on axial elongation, and whether such differences may be due to differences between the EGF family members in their affinity to the EGF receptor. The EGF receptor is activated by 7 different growth factors, including EGF, transforming growth factor-a, heparin binding EGF-like growth factor and betacellulin as high-affinity ligands, and epigen, epiregulin and amphiregulin as low-affinity ligands, binding about 10- to 100-fold more weakly than the high-affinity ligands [31]. In addition to the characteristics of receptor binding, studies have reported distinct EGF receptor-dependent cellular responses to the different ligands with respect to cell proliferation, differentiation and motility. The various EGF receptor ligands also qualitatively and quantitatively differ in the induction of the downstream signals [31]. It has thus remained unclear how the various EGF receptor ligands promote distinct cellular signaling responses through the same receptor tyrosine kinase. With respect to the observations made in our study, that betacellulin as a high-affinity ligand of the EGF receptor showed, when its antibody was applied, the weakest association with a reduction of axial elongation, may be due to the differences between the various EGF receptor ligands in their influence on the reaction of the cells (in terms of proliferation, differentiation and motility) and on the induction of the downstream signals.

Our study has several limitations which should be taken into account. First, it is an experimental study for which, as for any experimental study, it is a priori unclear, whether its findings can be transferred into a clinical setting. In particular, it has remained unaddressed and fully unclear, whether a potential clinical application of intravitreally injected antibodies of EGF family members may be useful for prevention of further axial elongation in highly myopic elderly patients and / or in patients in the school children age. Second, the number of guinea pigs in the groups was relatively small, so that an association between the intraocularly applied betacellulin might have become statistically clearer if more animals had been included. Third, we applied goggles with a refractive power of -10 diopters for induction of axial elongation, while in other studies, goggles with different refractive powers, such as -4 diopters were used. Fourth, we did not asses a dose-dependency of the effect of the EGF family members on axial elongation. Fifth, in addition to axial length, also the coronal diameters of the eyes could have been measured post mortem to obtain information about an eye enlargement in the horizontal and vertical direction. Sixth, histomorphometric measurements of the thickness of the retina, choroid, Bruch´s membrane and sclera would have added to the information provided by the study. Seventh, the removal of the goggles for performing the intravitreal injections might have had an influence on axial elongation. In a recent study by Benavente-Perez, removal of goggles for 30 min twice/day during a period of four weeks in marmosets undergoing lens-induced myopization led to a reduced axial elongation [32]. In our study however, the goggles were removed for less than 15 min once every 4 weeks, and the procedure was the same for the study group and the control group. It might therefore have been unlikely, that the short-term removal of the goggles once every 4 weeks had a major influence on the results of our study. Eighth, the differences between the study groups and the control groups were more marked for the measurements of axial length than for the readings of the vitreous cavity length. Reasons may have been a higher difficulty and a thus greater inaccuracy in measuring the vitreous cavity length. Ninth, partial coherence laser interferometry as compared to sonography might have been a more precise method to perform biometry to measure the ocular axial length and the vitreous cavity depth.

In conclusion, intraocularly applied antibodies against the EGF family members epiregulin, epigen, and to a minor degree, betacellulin, were associated with a reduction of lens-induced axial elongation in young guinea pigs. The observations fit with the notion of the EGF family members being involved in the process of axial elongation.

## Data Availability

All data generated or analysed during this study are included in this published article.
